# High Doses of Antidepressants and Long-term Treatment of Obsessive-compulsive Disorder: Another Barrier to Accessing Deep Brain Stimulation?

**DOI:** 10.31083/AP41734

**Published:** 2025-10-20

**Authors:** Emma Verstraete, Erwin Dreesen, Koen Schruers, Franciska AM Desplenter, Chris Bervoets

**Affiliations:** ^1^Department of Adult Psychiatry, University Psychiatric Center, Katholieke Universiteit Leuven, 3000 Leuven, Belgium; ^2^Clinical Pharmacology and Pharmacotherapy Unit, Department of Pharmaceutical and Pharmacological Sciences, Katholieke Universiteit Leuven, 3000 Leuven, Belgium; ^3^Department of Psychiatry, Maastricht University, 6211 LK Maastricht, The Netherlands; ^4^Top Clinical Center for Anxiety, OCD and Trauma, Mondriaan Mental Health Center, 6200 MD Maastricht, The Netherlands; ^5^Hospital Pharmacy, University Psychiatric Center, Katholieke Universiteit Leuven, 3070 Kortenberg, Belgium; ^6^Clinical Pharmacology and Pharmacotherapy, Department of Pharmaceutical and Pharmacological Sciences, Katholieke Universiteit Leuven, 3000 Leuven, Belgium; ^7^Leuven Brain Institute, Katholieke Universiteit Leuven, 3000 Leuven, Belgium

## 1. Introduction to Obsessive-compulsive Disorder

Obsessive-compulsive disorder (OCD) is a psychiatric condition characterized by intrusive thoughts (obsessions) and repetitive behaviors or mental acts (compulsions) that cause significant distress or impair functioning [1]. Neurobiologically, structural abnormalities are observed in OCD in the cortico-striato-thalamo-cortical circuits, including in the orbitofrontal cortex, anterior cingulate cortex, and striatum [2]. The median age of onset is 19 years, and in 60–70% of cases, the age of onset falls before the age of 25 years [3]. The lifetime prevalence of OCD is 2.3% in adults and the one-year prevalence is 1.3% [4]. The prevalence figures are similar in both sexes [1]. The majority of cases involve a comorbid psychiatric condition: 29–42% have major depressive disorder in addition to OCD, and 24–40% also have an anxiety disorder [5]. Psychological factors play a crucial role in the development and course of OCD. Adverse life experiences and environmental factors are closely related to symptom severity and treatment outcomes in individuals with OCD [6]. Seeking appropriate help for OCD often takes several years, ranging from 3.28–17 years after the onset of symptoms [7]. In the short term, OCD appears to have a chronic course with little chance of remission, however one prospective study found a remission rate of 42% after 15 years of follow-up [7].

## 2. Current Evidence-based Treatment

Treatment consists of cognitive behavioral therapy (CBT) combined with exposure-response prevention and/or pharmacotherapy, usually with selective serotonin reuptake inhibitors (SSRIs) [8]. SSRIs include fluoxetine, fluvoxamine, paroxetine, sertraline, citalopram and escitalopram, with the latter two not approved for OCD [9]. Clomipramine, a tricyclic antidepressant, is also registered. All these options are more effective than placebo, with no significant differences among them in efficacy [10]. Their serotonergic mechanism aligns with the hypothesis that OCD is based on a serotonergic dysfunction. The numbers needed to treat for SSRI monotherapy are 6.3 for low, 6.3 for medium, and 4.5 for high doses, reflecting a clinically meaningful effect [11]. The one third of patients who do not respond adequately to 12 weeks of SSRI treatment, experience significant symptom improvement with antipsychotic augmentation [12]. An umbrella review with meta-analysis found that CBT appeared to be more effective than was treatment with SSRIs. However, most studies evaluated the efficacy of CBT in patients who were on a stable dose of antidepressants [13]. The “gold standard” for measuring the severity of OCD symptomatology is the Yale-Brown Obsessive-Compulsive Scale (Y-BOCS) [4]. In OCD, a 25–35% decrease in the Y-BOCS score, compared with the pre-treatment score, is said to have the most reliable predictive value in terms of treatment response [14].

The American Psychiatric Association (APA) guideline, published in 2007, recommended an SSRI for at least 8 to 12 weeks, including 3 to 6 weeks at the maximum tolerable dose. If ineffective, switching to another SSRI or clomipramine (max. 250 mg/day) was advised, although partial responders should receive antipsychotic augmentation (e.g., risperidone) [9]. The guideline stated that there is insufficient evidence regarding success rate when applying a switching strategy. The National Institute for Health and Care Excellence (UK) has a similar guideline, published in 2005 [15]. Both guidelines are based on phase III clinical trials from the 1990s and early 2000s. With pharmacotherapy, up to 90% of cases have a clinically meaningful response on the Y-BOCS score [16].

## 3. Emerging Challenges in OCD Treatment

First, we consider the paradigm of high-dose SSRI treatment. A 2010 meta-analysis, which included 9 randomized, double-blind, placebo-controlled clinical trials comparing various fixed doses of SSRIs, demonstrated that high-doses of SSRI were significantly more effective in reducing OCD symptoms than were moderate or low doses [11]. However, there are two important limitations that should be considered regarding that analysis: (1) The included studies from the 1990s–2000s were limited in number, outdated, and many were subject to sponsorship bias, given the involvement of the pharmaceutical industry. (2) Although this meta-analysis indicated a statistically significant advantage of high-doses of SSRI over lower doses, concerns remained regarding the dropout rates in these studies. The authors concluded that dropout did not pose a significant statistical issue, but a meta-analysis from 2021 that excluded studies with high risk of bias, indicated that high dropout rates may compromise the clinical efficacy of high-dose treatment strategies [17]. That research proposed a 40-mg fluoxetine equivalent as a new target dose for SSRIs.

Second is the strategy of long-term high-dosing. A 2016 meta-analysis assessed the temporal course of SSRI-treatment effects [18]. That analysis found that statistically significant improvements in OCD symptoms could be observed as early as 2 weeks after treatment was initiated. By 6 weeks, over 75% of the mean improvement had been achieved. Moreover, between weeks 3 and 6 of treatment, a clinically meaningful difference in efficacy between high and low doses became evident. Notably, as with the 2010 meta-analysis, dropout rates were not adequately addressed. Those clinical data did not support the conventional wisdom, as stated by the APA as base for the guideline, that patients will not experience substantial improvement until 4–6 weeks after starting medication, and some may not show clear progress for up to 12 weeks.

Last, the current pharmacotherapeutic strategy warrants a critical stance regarding its pharmacodynamic rationale. Studies noted that dose escalation of an SSRI reaches a plateau in serotonin reuptake transporter (SERT) receptor occupancy of around 80–85%. Further dose escalation did not result in an additional serotonergic effect [19]. The plateau corresponds to the receptor occupancy achieved with the standard dose for depression treatment, equivalent to a low dose for OCD treatment. A positron emission tomography (PET) study of clomipramine showed that 80% receptor occupancy could already be obtained at a single 10 mg dose [20]. There were indications that lower doses might have been even more effective in terms of 5HT-receptor occupancy than were higher doses [19]. At high SSRI doses, other pharmacodynamic effects may influence efficacy and tolerability. For venlafaxine there is a known dose relation to noradrenergic effects, but high doses did not seem to be effective in patients with OCD [10], which does not fit the idea that noradrenergic-receptor mechanisms play a role. Paroxetine and clomipramine have relatively high noradrenaline-transporter affinity at standard dosing, and sertraline has high dopamine-transporter affinity [21]. Alternative pharmacodynamic effects at high doses are missing from product monographs. Our correspondence with pharmaceutical companies only provided the publicly available pharmacodynamics data, adding no new insights. The European Medicines Agency (EMA) publishes European Public Assessment Reports (EPAR) to promote transparency. However, for older national registrations, such as SSRI approvals, transparency requirements are less defined. Regulatory agencies like EMA and the U.S. Food and Drug Administration have mandated rational dose justification for marketed drugs. For EMA, this was guided by the International Council for Harmonisation guideline E4 (ICH E4) guideline (1994), based on the dose-finding approaches of that time [22], which sometimes resulted in higher doses than necessary. In 2005, EMA introduced a guideline addressing this issue, which required randomized controlled trial (RCTs) for OCD treatment to test three doses [23]. PET/single-photon emission computed tomography (SPECT) imaging and measurement of SERT occupancy have advanced over time [24]. Given this evolution, it is understandable that the ceiling effect of SERT occupancy was not yet recognized, leading to limited opposition to the dose-escalation strategy.

## 4. Treatment Resistance and the Role of Deep Brain Stimulation (DBS)

With pharmacotherapy, up to 90% of patients with OCD have at least a clinically meaningful response. For the remaining 10% with therapy resistance, if the symptoms present are severe, deep brain stimulation (DBS) may be considered. The criteria to qualify as a therapy-resistant patient for DBS are the following steps without result: two different SSRIs for 12 weeks at maximum dose; combining an SSRI with an antipsychotic; clomipramine for 12 weeks at maximum dose; and adequate CBT [25].

## 5. Potential Barriers to DBS

The inclusion criteria for DBS are conservative, which is reasonable in light of the higher risk of an invasive treatment, the debatable risk/benefit profile, and the social cost [26]. The criteria are based on the current pharmacological guidelines and switch-strategy. Considering the combined evidence on the efficacy of a long-term high-dose regimen and the pharmacodynamic rationale, these inclusion criteria warrant reevaluation. In clinical practice, OCD patients are often not prescribed the maximum recommended SSRI-doses [27]. Given recent findings on dropout rates in high-dose treatment [17], issues related to tolerance may contribute to this. Physicians with a cautious approach toward balancing side effects and therapeutic effects may fail to meet all the criteria for referring patients for DBS in non-responsive cases. Whether there is a crisis in accessing DBS remains subject to debate; the issue may lie in limited patient inflow and/or in infrastructural and institutional barriers [28]. Neurosurgeons have argued that psychiatrists insufficiently refer patients because of a lack of DBS knowledge. Our argument is that, in addition to this, the paradigm of long-term high-dose administration may also hinder DBS access, and that reevaluation of the current inclusion criteria could improve access to DBS.

## 6. Conclusion

In this paper, we examined the rationale of the current guideline for the pharmacological treatment of OCD and the inclusion criteria for DBS. It appears that higher doses of SRIs do not lead to increased serotonergic-receptor occupancy and that these high doses may involve other neurotransmitters. With high doses, there are higher dropout rates. In determining the target dose for an SSRI in OCD treatment, one should consider tolerability as well as effectiveness. The requirement for multiple 12-week high-dose SSRI trials before a patient is deemed treatment-resistant and eligible for DBS, lacks pharmacological and clinical justification.

We recommend updating the treatment guidelines for OCD with this new evidence, as a target dose and shorter treatment duration would lower the burden on the patient. Also, transparency regarding the studies underlying indication registration is crucial for advancing our understanding of the neurochemical basis of OCD. In clinical studies of OCD patients, exploring the differences between high and low doses, and short- and long-term treatment, are warranted. Although substantial evidence supporting the utility of therapeutic drug monitoring in OCD is lacking, measuring plasma levels of serotonin reuptake inhibitor (SRIs) and examining their relationship with therapeutic response could provide valuable insights [29]. In summary, a critical reflection on the existing treatment guidelines and non-response criteria is essential for improving treatment outcomes in OCD.

## Availability of Data and Materials

This study is based entirely on previously published literature. No new datasets were generated or analyzed. All sources used are cited in the reference list.
